# Supplementary material for: First Identification and Pathogenicity Evaluation of an EV-G17 Strain Carrying a Torovirus Papain-like Cysteine Protease (PLCP) Gene in China
Source: Viruses. 2023 Aug 15;15(8):1747. doi: 10.3390/v15081747 (PMC10459844; doi:10.3390/v15081747)
Supplement: Supplementary file 1 [file viruses-15-01747-s001.zip › viruses-2547187-supplementary.pdf]

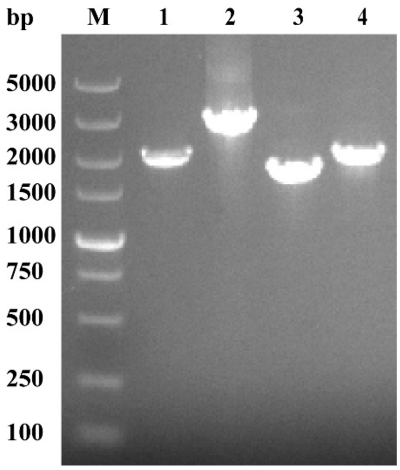

Figure S1. Amplification of the complete genome of EV-G/YN23/2022 isolate. Lane M, DNA Marker DL 5000. Lane 1 to 4, four overlapping PCR products of EV-G/YN23/2022 genome.

Table S1. Information on the 47 reference EVGs downloaded from GenBank.

Reference strains	Country/year	Accession No.	Reference strains	Country/year	Accession No.
UKG/410/73	GBR/2002	Y14459	744257	VIE/2012	KJ156451
Texas1	USA/2014	KY498016	To-4	JPN/2019	LC535380
Ishi-Ya4-1	JPN/2016	LC316789	Ishi-29	JPN/2019	LC535403
KNU-1811	KOR/2018	MH663501	714270/CaoLanh	VIE/2012	KT265903
EVG 02/NC_CHI	CHI/2014	MF782664	714405/CaoLanh	VIE/2012	KT265909
HgTa1	JPN/2016	LC316794	NIV-1740787	IND/2017	MN734577
LP54	GBR/2002	AF363455	724307/ChauThanh	VIE/2012	KT265941
HgYa2-1	JPN/2015	LC316791	BS14-173H2	VIE/2014	KT266010
Ishi-4	JPN/2018	LC535372	Ishi-10	JPN/2019	LC535396
Bu3-4	JPN/2014	LC316795	EVG 08/NC	USA/2015	KY761948
Ishi-Ka5-1	JPN/2016	LC316813	Ishi-Ya4	JPN/2017	LC549655
K23	HUN/2008	HQ702854	Kana-Ebi4	JPN/2018	LC549658
Iba464-3-2	PN/2015	LC316817	Kana-Ebi7	JPN/2019	LC549661
714361/CaoLanh	VIE/2012	KT265906	F26-2	GER/2013	MF113370
TB4-OEV	HUN/2009	JQ277724	GER/F8-2/04-02-2013	GER/2013	MF113372
HgTa2-1-2	JPN/2015	LC316819	JL14	CHI/2014	KU297674
PEV-B-KOR	KOR/2009	JQ818253	Texas2	USA/2014	KY498017
990/UK-NI	GBR/2018	MG958646	HgOg2-2	JPN/2015	LC316774
Ishi-3	JPN/2018	LC535369	Kana-Uchi13	JPN/2019	LC549657
Iba26-489	JPN/2014	LC316823	Iba26-506	JPN/2014	LC316792
734087/ThanhBinh	VIE/2012	KT265961	Ishi-24	JPN/2019	LC535402
Kana-Uchi4	JPN/2019	LC549656	Ishi-Ya3-2	JPN/2016	LC316825
Ishi-Im9-1	JPN/2016	LC316831	HgYa2-3-1	JPN2015	LC316827
724219/ChauThanh	VIE/2012	KT265931			

Table S2. The RT-qPCR primers used to measure mRNA levels of various cytokines.

Primer Name	Sequence (5'→3')	Reference
pISG15-F	GATCGGTGTGCCTGCCTTC	[36]
pISG15-R	CGTTGCTGCGACCCTTGT	
pIRF7-F	CTGCGATGGCTGGATGAA	[37]
pIRF7-R	TAAAGATGCGCGAGTCGGA	
pIFN-β-F	AGCAGATCTTCGGCATTCTC	[35]
pIFN-β-R	GTCATCCATCTGCCCATCAA	
pIFN-λ3-F	GTTCAAGTCTCTGTCCCCAC	
pIFN-λ3-R	GCTGCAGTTCCAGTCCTC	
βActin–F	CCCAGCACCATGAAGATCAA	[34]
βActin–R	GATCCACATCTGCTGGAAGG	
IFN-α-F3	TTCTGCACTGGACTGGATC	
IFN-α-R3	TCTGTGGAAGTATTTCCTCACAG	
IL-1β-F	ACCCAAAACCTGGACCTTGG	[32]
IL-1β-R	CATCACAGAAGGCCTGGGAG	
IL-6-F	CTCATTAAGTACATCCTCGG	
IL-6-R	GTCTCCTGATTGAACCCAGA	
pTNF-a-F	CCTACTGCACTTCGAGGTTATC	
pTNF-a-R	ACGGGCTTATCTGAGGTTTG	
IL-18-F	CGTGTTTGAGGATATGCCTGATT	[33]
IL-18-R	TGGTTACTGCCAGACCTCTAGTGA	

32. Zhou, P.; Li, L.F.; Zhang, K.; Wang, B.; Tang, L.; Li, M.; Wang, T.; Sun, Y.; Li, S.; Qiu, H.J. Deletion of the H240R gene of african swine fever virus decreases infectious progeny virus production due to aberrant virion morphogenesis and enhances inflammatory cytokine expression in porcine macrophages. *J Virol* **2022**, *96*, e0166721.
33. Razzuoli, E.; Mignone, G.; Lazzara, F.; Vencia, W.; Ferraris, M.; Masiello, L.; Vivaldi, B.; Ferrari, A.; Bozzetta, E.; Amadori, M. Impact of cadmium exposure on swine enterocytes. *Toxicol Lett* **2018**, *287*, 92-99.
34. Temeeyasen, G.; Sinha, A.; Gimenez-Lirola, L.G.; Zhang, J.Q.; Pineyro, P.E. Differential gene modulation of pattern-recognition receptor TLR and RIG-I-like and downstream mediators on intestinal mucosa of pigs infected with PEDV non S-INDEL and PEDV S-INDEL strains. *Virology* **2018**, *517*, 188-198.
35. Deng, X.; van Geelen, A.; Buckley, A.C.; O'Brien, A.; Pillatzki, A.; Lager, K.M.; Faaberg, K.S.; Baker, S.C. Coronavirus endoribonuclease activity in porcine epidemic diarrhea virus suppresses type i and type iii interferon responses. *J Virol* **2019**, *93*.
36. Yang, K.; Xue, Y.; Niu, H.; Shi, C.; Cheng, M.; Wang, J.; Zou, B.; Wang, J.; Niu, T.; Bao, M., *et al.* African swine fever virus mgf360-11l negatively regulates cgas-sting-mediated inhibition of type i interferon production. *Vet Res* **2022**, *53*, 7.
37. Xue, Q.; Liu, H.; Zhu, Z.; Yang, F.; Ma, L.; Cai, X.; Xue, Q.; Zheng, H. Seneca valley virus 3C^{pro} abrogates the IRF3- and IRF7-mediated innate immune response by degrading IRF 3 and IRF 7. *Virology* **2018**, *518*, 1-7.

Table S3. Information about the recombination event of EV-G/YN23/2022.

Recombinant Event	Breakpoints		Major (Similarity)	Minor (Similarity)	p-Value of the Detection Methods						
	**Beginning**	**Ending**			**RDP**	**GENECONV**	**BootScan**	**MaxChi**	**Chimaera**	**SiScan**	**3Seq**
1	845	3359	MF782644 (92.9%)	Unknow	1.766×10^{-54}	4.278×10^{-43}	3.906×10^{-44}	1.157×10^{-27}	8.523×10^{-31}	1.638×10^{-53}	4.353×10^{-11}
